# Multi-lineage reprogramming and NOTCH-associated signaling remodeling characterize the aggressive biology of early-onset colorectal cancer

**DOI:** 10.3389/fonc.2026.1962237

**Published:** 2026-10-22

**Authors:** Hongda Shi, Li Qiao, Qianqian Su, Hui Gao, Yang Cao

**Affiliations:** 1 Department of Gastrointestinal Surgery, Union Hospital, Tongji Medical College, Huazhong University of Science and Technology, Wuhan, China; 2 Department of Health Toxicology, Key Laboratory for Environment and Health, School of Public Health, Tongji Medical College, Huazhong University of Science and Technology, Wuhan, China; 3 Department of Chronic and Noncommunicable Disease Prevention and Control, Jiaxing Center for Disease Control and Prevention, Jiaxing, China

**Keywords:** cancer-associated fibroblasts, colorectal cancer, early-onset colorectal cancer, NOTCH signaling, tumor microenvironment

## Abstract

**Introduction:**

Early-onset colorectal cancer (EOCRC), defined as colorectal cancer diagnosed before 50 years of age, represents an emerging global health challenge with rapidly increasing incidence. Although EOCRC exhibits distinct clinical and molecular characteristics, the cellular ecosystem and mechanisms underlying its aggressive behavior remain incompletely understood.

**Methods:**

Here, we integrated seven independent single-cell RNA sequencing cohorts comprising 473,939 high-quality cells from 174 colorectal cancer (CRC) patients (39 EOCRC and 135 average-onset CRC [AOCRC]) to construct a comprehensive cellular landscape across age groups. After Harmony-based integration, nine major cellular lineages were identified, and malignant epithelial cells were characterized using chromosome-level copy number variation inference. We systematically compared malignant and stromal compartments, followed by malignant-state characterization, trajectory analysis, and cell -cell communication modeling using Slingshot, CellChat, and NicheNet.

**Results:**

EOCRC displayed extensive remodeling of the tumor ecosystem rather than isolated epithelial alterations. Malignant epithelial cells in EOCRC showed increased enrichment of inflammation-associated and Cycling (S/DNA damage response [DDR]) states. In parallel, proliferating endothelial cells and MKI67-positive proliferative cancer-associated fibroblasts were expanded in EOCRC, indicating stromal-state remodeling. Copy number variation analyses demonstrated that genomic instability differences between EOCRC and AOCRC were primarily explained by differential microsatellite instability (MSI) status distribution rather than age-related genomic alterations. Cell -cell communication analyses revealed selective remodeling of epithelial-stromal signaling networks, with recurrent involvement of NOTCH-associated mediators. NicheNet prioritized NOTCH-associated ligands (DLL4, JAG1) among candidate stromal-derived regulators of EOCRC transcriptional programs. A refined five-gene NOTCH-epithelial-mesenchymal transition (EMT) panel (HEY1, ZEB1, TWIST1, VIM, FN1) was associated with EOCRC-specific poor survival across two independent cohorts, and *in silico* perturbation further suggested that increased HEY1 activity may reinforce the proliferative state enriched in EOCRC.

**Discussion:**

Together, our study establishes EOCRC as a multicellular ecosystem characterized by coordinated malignant, stromal, and signaling reprogramming. These findings identify NOTCH signaling as a potential age-specific prognostic association that warrants functional validation in EOCRC.

## 1 Introduction

Colorectal cancer (CRC) remains the third most commonly diagnosed malignancy and the second leading cause of cancer-related mortality worldwide (1). While overall CRC incidence has declined in older adults, largely attributable to screening, the incidence of early-onset CRC (EOCRC), diagnosed before age 50, has risen at 2%–4% annually over the past 3 decades in high-income countries (2–4). This epidemiologic shift is projected to continue; by 2030, EOCRC is expected to account for approximately 11% of colon cancers and 23% of rectal cancers in the United States (5). Increasing evidence indicates that EOCRC is not simply an earlier manifestation of average-onset colorectal cancer (AOCRC), but rather represents a biologically distinct disease entity with unique molecular and clinical characteristics. Integrated genomic and epigenomic studies have revealed age-associated alterations in EOCRC, including distinct mutational patterns, DNA methylation landscapes, and non-coding RNA signatures, suggesting divergent evolutionary trajectories compared with AOCRC (6). However, these molecular alterations alone cannot fully explain the aggressive clinical behavior of EOCRC, including advanced disease presentation and poorer outcomes observed in subsets of young patients. The cellular programs and tumor ecosystem mechanisms that translate molecular differences into age-associated tumor phenotypes remain incompletely understood.

Although inherited predisposition syndromes contribute to a proportion of EOCRC cases, the majority of patients develop disease without identifiable hereditary causes, highlighting the importance of tumor-intrinsic and microenvironmental mechanisms in shaping EOCRC biology (7). The tumor microenvironment (TME) is increasingly recognized as an active regulatory ecosystem composed of malignant cells, immune populations, stromal cells, endothelial cells, and extracellular matrix components. Through dynamic cellular interactions and signaling exchanges, the TME regulates tumor initiation, malignant progression, angiogenesis, immune evasion, and therapeutic response (8, 9). Therefore, understanding how the cellular composition and communication networks of the TME differ between EOCRC and AOCRC may provide critical insights into the biological basis of early-onset disease.

Recent advances in single-cell RNA sequencing (scRNA-seq) have enabled high-resolution characterization of cellular heterogeneity and molecular states within CRC. Previous studies have identified diverse malignant epithelial states, immune microenvironmental architectures, and dynamic cellular transitions associated with CRC progression (10, 11). Furthermore, single-cell analyses have demonstrated that stromal populations, including cancer-associated fibroblasts (CAFs) and endothelial cells (ECs), consist of functionally distinct subsets that contribute to tumor evolution through regulation of extracellular matrix remodeling, angiogenesis, immune modulation, and therapy resistance (12–14). However, most existing single-cell studies have focused on conventional CRC populations, whereas the cellular ecosystem of EOCRC remains largely unexplored. In particular, whether EOCRC is characterized by unique malignant epithelial states, distinct stromal remodeling patterns, or specific intercellular communication networks that promote aggressive tumor behavior remains unknown. Addressing these questions requires an integrated single-cell framework capable of simultaneously resolving malignant cell evolution, stromal heterogeneity, and intercellular communication across different age groups.

Here, we generated a comprehensive single-cell atlas of EOCRC by integrating seven independent scRNA-seq cohorts comprising 473,939 high-quality cells from 174 CRC patients, including 39 EOCRC and 135 AOCRC cases. Through systematic comparison of malignant epithelial states, stromal remodeling, copy number variation (CNV) landscapes, developmental trajectories, and intercellular signaling networks, we identify coordinated multi-lineage reprogramming associated with EOCRC biology. Our analyses reveal that proliferative malignant epithelial state transitions, expansion of activated stromal populations, and NOTCH-associated communication circuits between tumor and microenvironmental compartments characterize EOCRC. Furthermore, we establish the clinical relevance of NOTCH signaling alterations through independent survival validation in The Cancer Genome Atlas Colon Adenocarcinoma and Rectum Adenocarcinoma (TCGA-COADREAD) cohort. Together, our study provides a high-resolution framework for understanding the cellular mechanisms underlying EOCRC aggressiveness and identifies potential age-specific prognostic associations warranting functional validation.

## 2 Materials and methods

### 2.1 Study design and data collection

Publicly available scRNA-seq datasets from CRC patients were collected and integrated for this study. The datasets included GSE132465, GSE178341, GSE188711, GSE205506, GSE236581, CNP0004138, and SYN26844071 (10, 15–20). Based on the age at diagnosis, the 174 included patients were classified into two groups: 39 EOCRC patients (<50 years) and 135 AOCRC patients (≥50 years). All datasets were obtained from publicly accessible repositories with appropriate ethical approvals. Clinical characteristics, including age, sex, tumor stage, tumor sidedness, and microsatellite instability (MSI) status, were extracted when available.

### 2.2 ScRNA-seq analysis

The read count matrices were processed using the R package Seurat (version 5.4.0). Low-quality cells were filtered using the following criteria: cells with fewer than 200 detected genes, fewer than 800 unique molecular identifiers, >10% mitochondrial reads, or potential doublets identified by DoubletFinder were excluded. Following quality control, the count matrix was log-normalized and scaled to a total of 10,000. The top 2,000 highly variable genes were used for principal component analysis. To correct for batch effects across different datasets, the top 20 principal components were integrated using Harmony (21). Batch effect correction quality was quantitatively assessed using the k-nearest-neighbor batch effect test (kBET) acceptance rate and batch mixing entropy, along with per-batch uniform manifold approximation and projection (UMAP) visualization and cell type composition comparison. Dimensionality reduction was performed using UMAP.

Unsupervised clustering was used to identify cell populations across the integrated dataset. Clusters were annotated on the basis of canonical marker genes and differential expression analysis. Major cellular lineages included epithelial cells, ECs, fibroblasts, pericytes, myeloid cells, T cells, B cells, plasma cells, and mast cells.

### 2.3 Malignant epithelial cell analysis

Epithelial cells were extracted from the integrated single-cell dataset for malignant cell identification. CNV profiles were inferred using fastCNV (version 1.1.4; 22), with immune and stromal cell populations (T cells, B cells, plasma cells, myeloid cells, fibroblasts, ECs, pericytes, and mast cells) serving as the normal reference. Malignant cells were identified by K-means clustering (k = 7) of chromosome arm-level CNV features (p and q arms for chromosomes 1–22 and X, 46 features total) combined with the overall CNV fraction (cnv_fraction) metric. Cells assigned to non-normal-like clusters were classified as malignant. The number of clusters (k = 7) was chosen to balance capture of distinct CNV archetypes against over-fragmentation; the malignant-versus-normal assignment was stable across alternative cluster counts (k = 5–9, **Supplementary Figure S3**). CNV burden was quantified using six complementary metrics: mean CNV fraction, percentage of genome altered (PGA), weighted genome instability index (wGII), number of arms gained, number of arms lost, and total aneuploidy score. Consensus clustering of per-patient arm-level CNV profiles was performed with resampling-based stability assessment. Per-arm CNV gain and loss event frequencies were compared between age groups using Fisher’s exact test with Benjamini–Hochberg correction (92 tests: 46 arms × 2 event types). Multivariate linear regression adjusting for stage, MSI status, tumor sidedness, and sex was used to assess the independent contribution of age group to CNV burden.

### 2.4 Characterization of functional states of cancer cells

Gene signatures representing functional states of cancer cells, including stemness, invasion, metastasis, proliferation, angiogenesis, apoptosis, cell cycle, DNA damage, DNA repair, hypoxia, inflammation, and quiescence, were curated from the CancerSEA database and literature. The irGSEA package (version 2.0.0; 23) was used to compute enrichment scores via AUCell, UCell, singscore, and single-sample gene set enrichment analysis (ssGSEA) methods, with results integrated by robust rank aggregation (RRA).

### 2.5 Cell–cell communication analysis

Cell–cell communication was inferred using CellChat (version 2.1.2; 24, 25). The number and strength of interactions among major cell populations were calculated to compare communication networks between EOCRC and AOCRC. To ensure comparability of interaction metrics, the AOCRC group was proportionally down-sampled to match the cell count of the EOCRC group, with cell type proportions preserved during down-sampling (documented in the analysis script). Key signaling pathways were analyzed, including NOTCH, WNT, TGFβ, VEGF, ANGPT, FGF, and IGF.

To further identify candidate ligands driving EOCRC-specific transcriptional programs, NicheNet analysis using the nichenetr package (version 2.2.0; 26) was performed. Genes upregulated in EOCRC relative to AOCRC within receiver cell populations were defined as the gene set of interest. The Pearson correlation coefficient between ligand expression and the regulatory potential score for downstream target genes prioritized ligand activity. We note that the NicheNet ligand candidate set encompasses secreted factors, extracellular matrix components, and cell-surface molecules; accordingly, the term “ligand” is used here broadly to refer to all prioritized signaling mediators, and only a subset (for example, DLL1, JAG1, and FGF2) are canonical secreted ligands.

### 2.6 Functional enrichment analysis

Gene Ontology (GO) biological process and Kyoto Encyclopedia of Genes and Genomes (KEGG) pathway enrichment of the 33 NicheNet target genes was performed using clusterProfiler, with the human genome as background (27). Terms were considered significant at Benjamini–Hochberg-adjusted P <0.05, and the top enriched terms were visualized as dot plots (fold enrichment versus gene count, colored by adjusted P-value).

### 2.7 Pseudotime trajectory analysis

Pseudotime trajectory inference was performed using Slingshot (version 2.10.0) with the malignant epithelial UMAP embedding as the input dimensionality reduction. The Stem-like population was designated as the trajectory root based on its highest stemness-marker expression among the nine malignant subtypes, and lineage curves were fitted through the malignant subtype clusters. The Kolmogorov–Smirnov test was used to compare pseudotime distributions between EOCRC and AOCRC at the cell level; to avoid pseudoreplication from treating individual cells as independent observations, pseudotime was additionally aggregated to the patient level (median per patient) and compared with the Wilcoxon rank-sum test.

### 2.8 Survival analysis

Overall survival (OS) was defined as the time from diagnosis to death from any cause. Survival analyses were performed using the TCGA-COADREAD cohort (n = 620) and GSE39582 cohort (n = 585) (28). To derive a robust prognostic panel, the 33 NicheNet-prioritized target genes were ranked by the strength of their age-specific interaction (gene × age group in Cox models, evaluated in both the TCGA-COADREAD cohort and an independent GSE39582 cohort), and the five genes with the strongest consistent interaction were retained (HEY1, ZEB1, TWIST1, VIM, FN1). The panel score was computed as the mean z-score across these five genes, and its prognostic value and age interaction were assessed in both cohorts with adjustment for stage, sex, and MSI status. All survival analyses were performed using the R packages “survival” (version 3.8-3) and “survminer” (version 0.4.9).

### 2.9 *In silico* perturbation of NOTCH signaling

To assess the functional contribution of NOTCH signaling to malignant epithelial states, *in silico* perturbation was performed using CellOracle (version 0.20.0; 29). A gene regulatory network was inferred from the malignant epithelial cells using the base GRN, and HEY1, the NOTCH downstream effector retained in the prognostic panel, was perturbed by setting its expression to 0 (knockout) or to an elevated value (overexpression). The resulting embedding shift was projected onto a proliferative–differentiated axis, defined by the centroid difference between the Cycling (S/DNA damage response (DDR))/Inflammation-associated and oxidative phosphorylation (OXPHOS)-high/Goblet/Secretory states, to obtain a signed directional shift per malignant subtype.

### 2.10 Statistical analysis

Cell subtype composition was compared between EOCRC and AOCRC at the patient level. For each patient, the number of cells in each subtype and the total number of cells were calculated to obtain subtype proportions. Differences in subtype proportions were assessed using quasibinomial generalized linear models (GLMs) with adjustment for clinical covariates (stage, sex, sidedness, and MSI status). All reported *P*-values were adjusted for multiple testing using the Benjamini–Hochberg (BH) procedure across all comparisons within each analysis. Throughout the manuscript, the term “significant” is used exclusively for comparisons where BH-adjusted *P* < 0.05. All box plots display individual patient data points, with the box representing median and interquartile range (IQR) and whiskers extending to 1.5 × IQR. All analyses were performed using R (version 4.5.1).

## 3 Results

### 3.1 A single-cell transcriptomic atlas reveals multi-lineage compositional shifts in EOCRC

To characterize age-associated cellular heterogeneity in CRC, we integrated seven publicly available scRNA-seq datasets encompassing 473,939 high-quality cells from 174 patients (39 EOCRC and 135 AOCRC; **Figure 1A**). Following batch correction using Harmony, integration quality was assessed by quantitative kBET analysis and per-batch UMAP visualization, demonstrating effective removal of dataset-specific batch effects while preserving biological variation (**Supplementary Figure S1**). Unsupervised clustering and cell type annotation identified nine major cellular compartments within the CRC TME, including epithelial cells, ECs, fibroblasts, pericytes, myeloid cells, T cells, B cells, plasma cells, and mast cells (**Figure 1B**). Cell identities were further confirmed by marker gene expression, with epithelial cells characterized by EPCAM/KRT18 expression, fibroblasts by DCN/LUM expression, ECs by PECAM1/VWF expression, immune populations by lineage-specific markers including CD3D/CD3E (T cells), CD79A/MS4A1 (B cells), CD68/LYZ (myeloid cells), and TPSAB1/CPA3 (mast cells) (**Figure 1C**). Although EOCRC and AOCRC shared these lineages, their cellular compositions differed significantly. EOCRC tumors displayed a significantly increased proportion of epithelial cells compared with AOCRC (*P* < 0.05), suggesting a greater enrichment of tumor epithelial components in younger patients. Conversely, EOCRC showed reduced stromal cell representation, including decreased fibroblast and EC fractions, whereas immune cell compartments, including T cells, B cells, plasma cells, myeloid cells, and mast cells, exhibited relatively comparable distributions between the two groups (**Figure 1D**). These findings indicate that EOCRC is characterized by an altered balance between malignant epithelial and stromal compartments, suggesting age-dependent differences in tumor architecture and microenvironmental organization.

**Figure 1:**
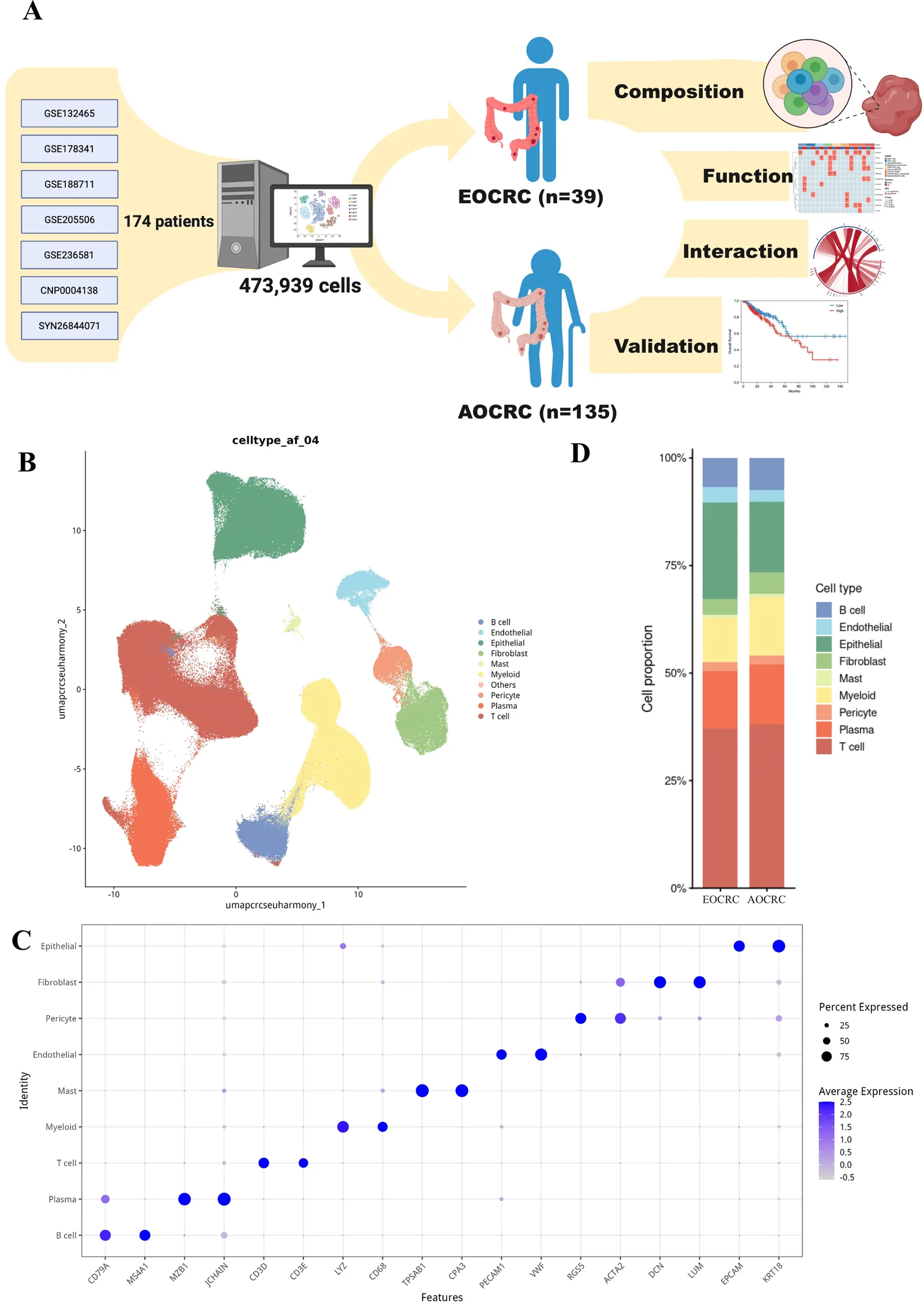
Study design and single-cell landscape of EOCRC and AOCRC. **(A)** Study overview and cohort composition. Seven publicly available scRNA-seq datasets were integrated, yielding 473,939 high-quality cells from 174 CRC patients (39 EOCRC and 135 AOCRC). **(B)** UMAP visualization of the integrated dataset colored by nine major cell lineages: epithelial cells, ECs, fibroblasts, pericytes, myeloid cells, T cells, B cells, plasma cells, and mast cells. **(C)** Dot plot showing representative marker genes across the major cell types. **(D)** Stacked bar plot showing the relative proportions of major cell types in EOCRC and AOCRC.

### 3.2 Malignant epithelial cell identification and CNV landscape characterization

Given that epithelial cells are the primary drivers of CRC behavior, the epithelial lineage was subclustered for high-resolution analysis. To distinguish malignant from normal epithelial cells, CNV profiles were inferred using fastCNV across 174 patients, with immune and stromal cell populations (T cells, B cells, plasma cells, myeloid cells, fibroblasts, ECs, pericytes, and mast cells) serving as the normal diploid reference, were applied. K-means clustering (k = 7) of 46 chromosome arm-level CNV features identified non-normal-like clusters as malignant, yielding 52,791 malignant epithelial cells from 166 patients (37 EOCRC and 129 AOCRC, **Figure 2A**). The remaining epithelial cells classified as CNV-normal (n = 17,843) were excluded from downstream malignant cell analyses.

**Figure 2:**
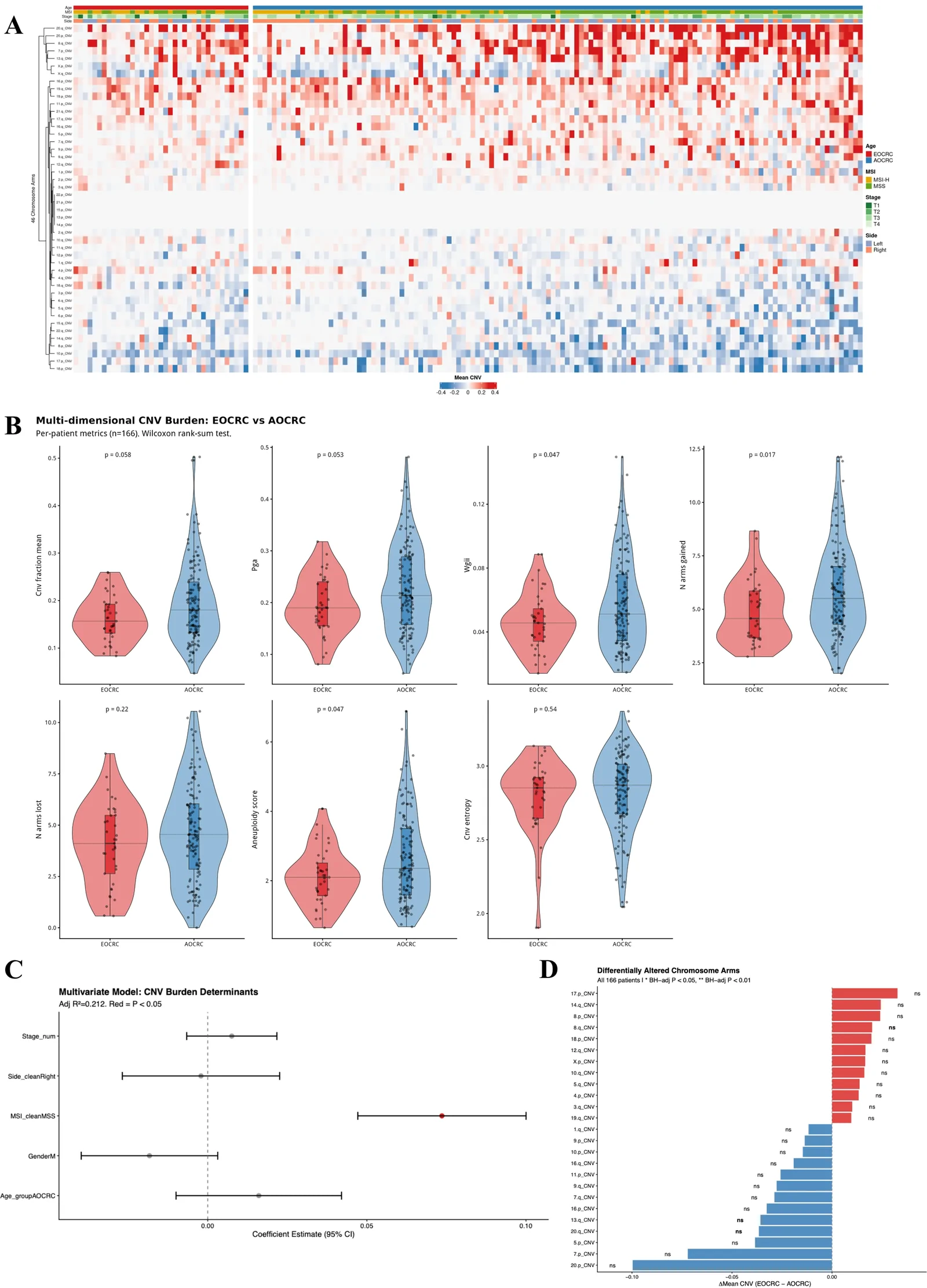
Malignant epithelial cell identification and CNV landscape characterization. **(A)** fastCNV-based malignant cell identification. UMAP of epithelial cells colored by K-means cluster (k = 7). Non-normal-like clusters were designated as malignant, yielding 52,791 malignant epithelial cells from 166 patients (37 EOCRC and 129 AOCRC). **(B)** Multi-dimensional CNV burden comparison between EOCRC and AOCRC at the patient level. Six complementary metrics are shown: mean CNV fraction, PGA, wGII, number of arms gained, number of arms lost, and total aneuploidy score. Box plots with individual patient data points. P-values from Wilcoxon rank-sum test. **(C)** Multivariate linear regression forest plot. Coefficient estimates and 95% confidence intervals for clinical covariates predicting per-patient mean CNV burden. Red, P <0.05. Adj R² annotated. **(D)** Chromosome arm-level CNV differential analysis. Top differentially altered chromosome arms ranked by |EOCRC-AOCRC| mean CNV difference. No arm reached BH-adjusted significance after correction for 92 tests (46 arms × 2 event types). See also **Supplementary Figure S2**.

With malignant cell identity established, we next characterized the genomic instability landscape of EOCRC versus AOCRC. Per-patient CNV burden was quantified using six complementary metrics: mean CNV fraction, PGA, wGII, number of arms gained, number of arms lost, and total aneuploidy score (**Figure 2B**). Multidimensional comparison revealed that three metrics reached nominal significance: AOCRC tumors exhibited higher wGII (*P* = 0.047), higher aneuploidy score (*P* = 0.047), and more gained chromosome arms (*P* = 0.017), while mean CNV fraction and PGA showed consistent directional trends (*P* = 0.058 and *P* = 0.053, respectively).

To determine whether these differences were attributable to age-of-onset or confounded by clinical covariates, we performed multivariate linear regression adjusting for stage, MSI status, tumor sidedness, and sex. This analysis revealed that MSI status, not age group, was the dominant determinant of CNV burden (microsatellite stable (MSS) vs. microsatellite instability-high: *β* = 0.074, *P* = 1.8 × 10^-7^; **Figure 2C**). After adjustment for MSI, the age-group effect was markedly attenuated and non-significant (*P* = 0.23). Per-arm gain and loss frequency analysis identified several chromosome arms with nominally significant age-group differences, but no individual arm reached BH-adjusted significance (**Figure 2D**). Sensitivity analyses stratified by MSI status, clinical stage, and tumor sidedness confirmed that EOCRC–AOCRC CNV differences were not robust to stratification (**Supplementary Figure S2**).

Taken together, these results demonstrate that EOCRC and AOCRC share broadly similar genomic instability landscapes once MSI status is accounted for. The apparent CNV differences between age groups are predominantly driven by the differential prevalence of MSI-H tumors, which characteristically harbor lower CNV burden and are enriched in the older-onset population. This finding has a critical implication: the aggressive epithelial phenotypes previously associated with EOCRC cannot be attributed to higher chromosomal instability. We therefore turned to transcriptional subtyping of the 52,791 CNV-confirmed malignant epithelial cells to identify the molecular basis of EOCRC’s biological distinctiveness.

### 3.3 EOCRC malignant epithelium exhibits distinct transcriptional states and age-associated compositional differences

Following malignant-cell identification by the CNV-inference approach, malignant epithelial cells were extracted and subjected to high-resolution reclustering. UMAP visualization resolved nine transcriptionally distinct malignant epithelial states: Stem-like, UDP-glucuronosyltransferase (UGT)-high, Goblet/Secretory, Metastasis-associated, OXPHOS-high, Cycling (S/DDR), Cycling (G2M), Inflammation-associated, and Neuroendocrine-like populations (**Figure 3A**). The transcriptional identities of these malignant epithelial states were supported by their cluster-specific gene-expression patterns (**Figure 3B**). The Metastasis-associated population expressed invasion- and tumor-progression-related genes, including MMP7 and S100A2, whereas the two cycling populations showed elevated expression of proliferation and cell-cycle genes such as MKI67, TOP2A, and CCNB1. Secretory and epithelial differentiation programs characterized Goblet/Secretory cells, while OXPHOS-high cells preferentially expressed genes associated with mitochondrial oxidative metabolism. Inflammation-associated cells displayed increased expression of inflammatory-response-related genes, supporting the functional annotation of the identified epithelial states.

**Figure 3:**
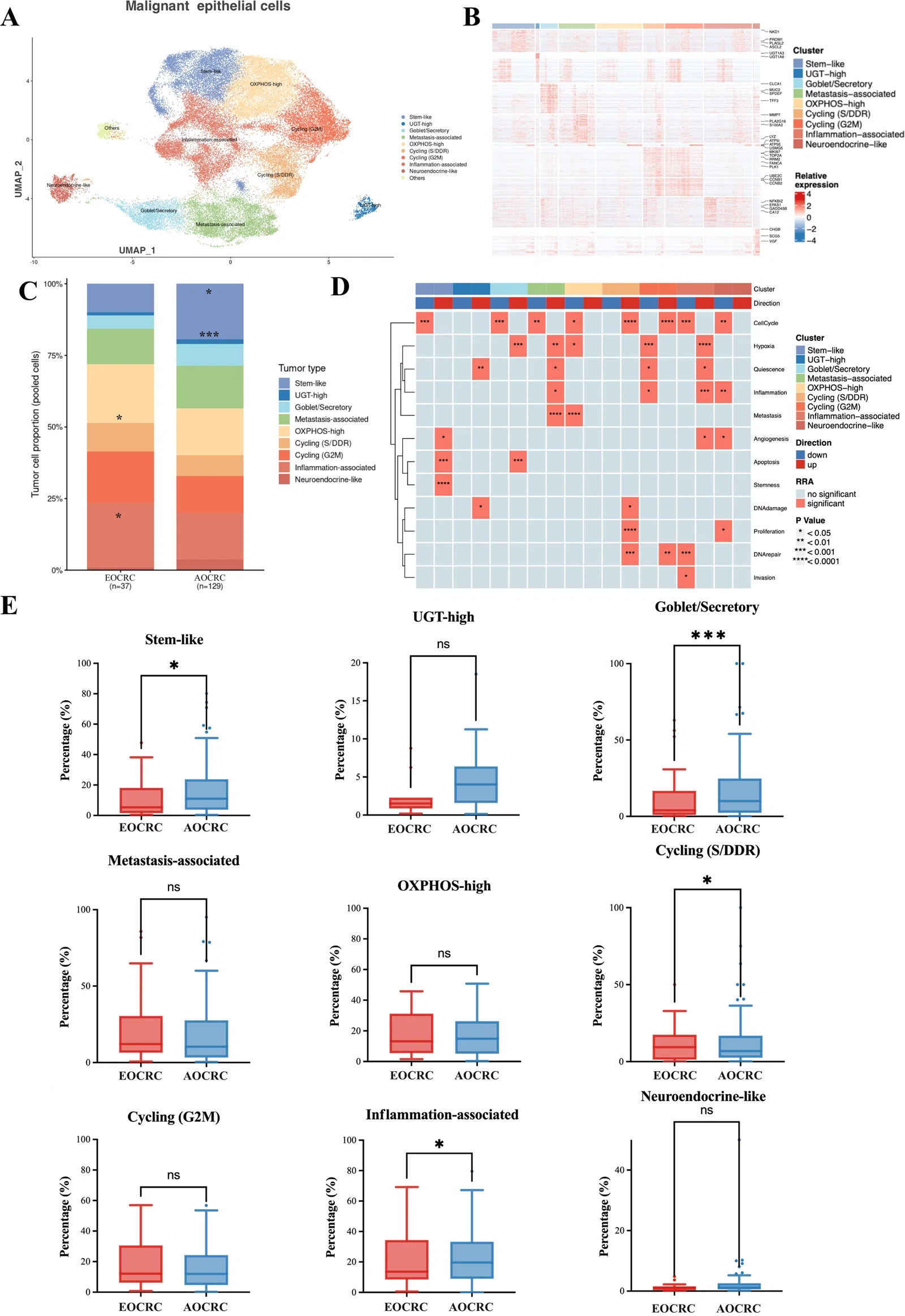
Distinct malignant epithelial states in EOCRC and AOCRC. **(A)** UMAP projection of the reclustered malignant epithelial cells. **(B)** Heatmap showing the expression of representative marker genes across the identified subclusters. **(C)** Stacked bar plot comparing the relative cellular proportions of these states between EOCRC and AOCRC. **(D)** Functional enrichment analysis (RRA) depicting the activation of specific biological programs across different clusters. **(E)** Comparison of the proportions of malignant epithelial subtypes between EOCRC and AOCRC at the patient level. Box plots illustrate the distribution of subtype proportions between the two groups. *P <0.05, **P <0.01, ***P <0.001, ****P <0.0001.

Comparison of the pooled malignant epithelial composition showed differences in the relative abundance of several cell states between EOCRC and AOCRC (**Figure 3C**), and patient-level proportions were subsequently compared using a quasibinomial GLM adjusting for stage, MSI status, tumor sidedness, and sex (**Figure 3E**). EOCRC tumors were characterized by a significant enrichment of inflammation-associated and Cycling (S/DDR) populations (*P* < 0.05). In contrast, AOCRC tumors displayed a higher proportion of OXPHOS-high and Goblet/Secretory malignant subtypes, consistent with a more differentiated and metabolically quiescent phenotype. To confirm that these age-associated compositional differences were not driven by any single cohort, we performed a leave-one-cohort-out analysis: after sequential removal of each of the seven datasets, the EOCRC enrichment of inflammation-associated and Cycling (S/DDR) states remained significant (*P* < 0.05 in six of seven iterations), and the corresponding covariate-adjusted odds ratios remained directionally consistent.

Functional enrichment analysis (RRA) further elucidated that the epithelial subclusters more prevalent in EOCRC were fundamentally wired for heightened clinical aggressiveness (**Figure 2D**). Specifically, the Cycling (S/DDR) and Inflammation-associated populations exhibited prominent enrichment for DNA damage response, cell cycle, and proliferation gene signatures, while OXPHOS-high and Goblet/Secretory subtypes, enriched in AOCRC, showed activation of metabolic and differentiation programs.

### 3.4 Pseudotime analysis reveals distinct differentiation trajectories and age-associated shifts in malignant epithelial states

To delineate the developmental relationships among malignant epithelial subtypes, pseudotime trajectories were inferred using Slingshot based on the UMAP embedding, with the Stem-like population specified as the root state. Three principal trajectories emerged from the Stem-like compartment. They extended toward distinct epithelial states, indicating multiple potential routes of malignant epithelial differentiation (**Figure 4A**). At the cell level, EOCRC cells were nominally enriched at intermediate pseudotime positions whereas AOCRC cells were relatively more represented at later positions; however, this apparent difference was not confirmed when pseudotime was aggregated at the patient level (median pseudotime per patient, Wilcoxon *P* = 0.725) (**Figures 4B, C**). Comparison across malignant epithelial subtypes further revealed a sequential organization of cell states along pseudotime (**Figure 4D**). Stem-like cells were concentrated at the earliest pseudotime positions, consistent with their designation as the trajectory origin. Inflammation-associated and OXPHOS-high cells predominantly occupied early-to-intermediate positions, whereas Cycling (G2/M cell-cycle transition (G2M)) and Cycling (S/DDR) cells were located mainly at intermediate pseudotime states. Metastasis-associated cells appeared at relatively advanced positions, while Goblet/Secretory, UGT-high, and Neuroendocrine-like cells were concentrated toward the latest pseudotime regions. Collectively, these results reveal heterogeneous differentiation trajectories within the malignant epithelial compartment; however, patient-level analysis did not confirm a significant age-associated shift in the distribution of these states.

**Figure 4:**
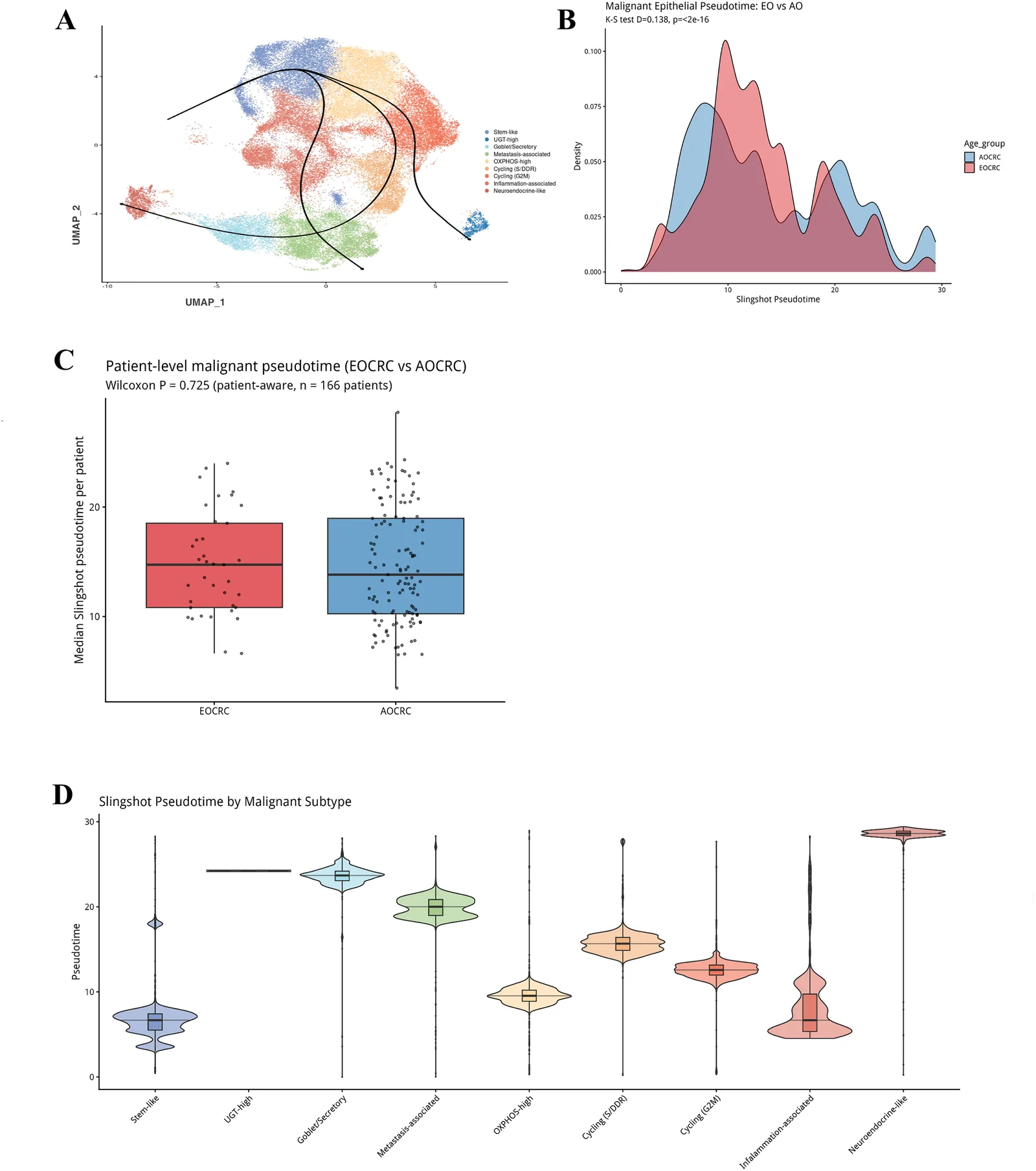
Slingshot pseudotime trajectory analysis of malignant epithelial differentiation. **(A)** UMAP of malignant epithelial cells overlaid with three Slingshot trajectory curves (black arrows), all originating from the Stem-like population. Cells colored by malignant subtype. **(B)** Pseudotime density distribution comparing EOCRC versus AOCRC. Kolmogorov–Smirnov test D statistic and P-value annotated. **(C)** Patient-level comparison of malignant epithelial pseudotime between EOCRC and AOCRC. For each patient, the median pseudotime value of malignant epithelial cells was calculated, and differences between age groups were assessed using the Wilcoxon rank-sum test. **(D)** Pseudotime distribution by malignant epithelial subtype (violin plot), showing the developmental progression from Stem-like through Cycling subtypes toward terminal differentiation states.

### 3.5 Remodeled endothelial and fibroblast niches underpin the heightened aggressiveness of EOCRC

After the characterization of malignant epithelial heterogeneity, age-associated alterations within the stromal microenvironment were further investigated. Endothelial and fibroblast/smooth muscle cell (SMC) compartments were independently extracted and subjected to high-resolution clustering to identify transcriptionally distinct cellular states.

Sub-clustering of ECs identified multiple vascular endothelial states, including high endothelial venule (HEV)-like vein endothelial cells, lymphatic endothelial cells, arterial endothelial cells, capillary endothelial cells, Tip endothelial cells, interferon (IFN)-activated endothelial cells, and proliferating/cycling endothelial cells (**Figure 5A**). These endothelial populations displayed distinct transcriptional profiles, reflecting functional diversity within the CRC vascular niche. Marker gene analysis further supported the annotation of endothelial subtypes (**Figure 5B**), with proliferating/cycling endothelial cells characterized by elevated expression of cell-cycle-associated genes, including MKI67, TOP2A, and CCNB2, whereas Tip ECs exhibited expression patterns associated with angiogenic activation, including PGF, ESM1, and ANGPT2. Comparison of EC compositions between EOCRC and AOCRC revealed significant age-associated differences in vascular states (**Figures 5B, C**). EOCRC tumors showed a higher proportion of proliferating/cycling and capillary ECs (*P* < 0.05), indicating increased representation of actively proliferating vascular cells. In contrast, AOCRC tumors displayed increased proportions of arterial ECs (*P* < 0.01), suggesting differences in vascular cellular organization between the two age groups. These findings indicate that EOCRC endothelial niches exhibit a distinct remodeling pattern characterized by increased proliferative endothelial states.

**Figure 5:**
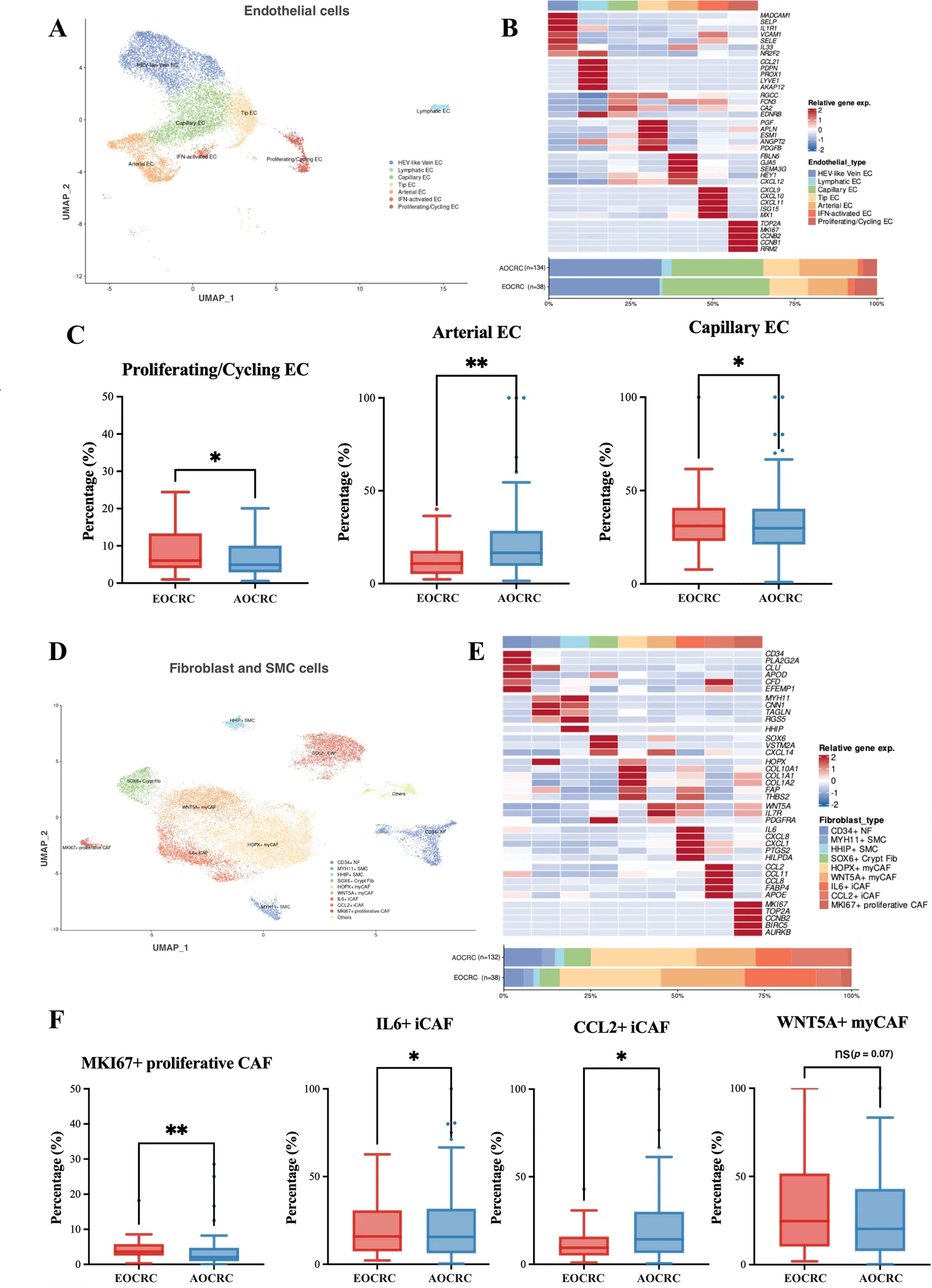
Endothelial and fibroblast lineage remodeling across EOCRC and AOCRC. **(A)** UMAP projection of the identified endothelial subclusters. **(B)** Heatmap showing the expression of representative marker genes across these endothelial subclusters. **(C)** Box plots quantifying the proportions of endothelial subclusters at the individual patient level. **(D)** UMAP projection of fibroblast and smooth muscle cell subclusters. **(E)** Heatmap showing the expression of representative marker genes across fibroblast and SMC subclusters. **(F)** Box plots quantifying the proportions of stromal subclusters at the individual patient level. ns, not significant, *P <0.05, **P <0.01.

The fibroblast and SMC compartments also demonstrated substantial transcriptional heterogeneity. Reclustering identified normal fibroblast populations, SMC populations, and multiple CAF states, including inflammatory CAFs (iCAFs), myofibroblast-like CAFs (myCAFs), and proliferative CAFs (**Figure 5D**). Expression profiling confirmed the identity of these stromal populations, with iCAF subsets characterized by cytokine-related programs (IL6 or CCL2), myCAF populations showing extracellular matrix-associated signatures (COL1A1, WNT5A), and proliferative CAFs exhibiting elevated expression of cell-cycle genes including MKI67, TOP2A, and AURKB (**Figure 5E**). Patient-level compositional analysis revealed significant differences in CAF states between EOCRC and AOCRC (**Figures 5E, F**). EOCRC tumors showed a significant increase in MKI67-positive proliferative CAFs (P <0.01), indicating enhanced representation of proliferative stromal populations. In addition, IL6-positive iCAFs displayed a higher proportion in EOCRC, whereas CCL2-positive iCAFs were relatively enriched in AOCRC (*P* < 0.05). The WNT5A-positive myCAF population showed a tendency toward increased abundance in EOCRC, although this difference did not reach statistical significance (*P* = 0.07).

Together, these results demonstrate that EOCRC is associated with coordinated remodeling of endothelial and fibroblast compartments, characterized by expansion of proliferative ECs and proliferative/inflammatory CAF states. These age-associated stromal alterations suggest distinct microenvironmental configurations between EOCRC and AOCRC.

### 3.6 Age-associated bidirectional signaling networks connect malignant epithelial cells with stromal and endothelial compartments

To characterize age-associated differences in intercellular communication, ligand–receptor networks were inferred across malignant epithelial, endothelial, and fibroblast populations. Differential interaction analysis revealed that specific communication routes involving malignant epithelial cells, CAFs, and endothelial populations differed between the two age groups (**Figure 6A**). Nevertheless, global CellChat analysis showed that AOCRC contained a greater total number of inferred interactions and higher aggregate interaction strength than EOCRC (**Figure 6B**). Thus, EOCRC was not characterized by a globally stronger communication network, but rather by selective remodeling of particular epithelial-stromal signaling relationships. To evaluate the donor- and cohort-level robustness of this inference, we repeated the CellChat analysis after balancing the number of EOCRC and AOCRC donors within each leave-one-cohort-out fold (**Supplementary Figure S4A**). After normalizing to the number of detected pathways, NOTCH relative information flow was comparable between EOCRC and AOCRC (0.47% vs 0.47%) and NOTCH remained among the top pathways in both age groups in all folds, indicating that NOTCH signaling is not specifically elevated in EOCRC.

**Figure 6:**
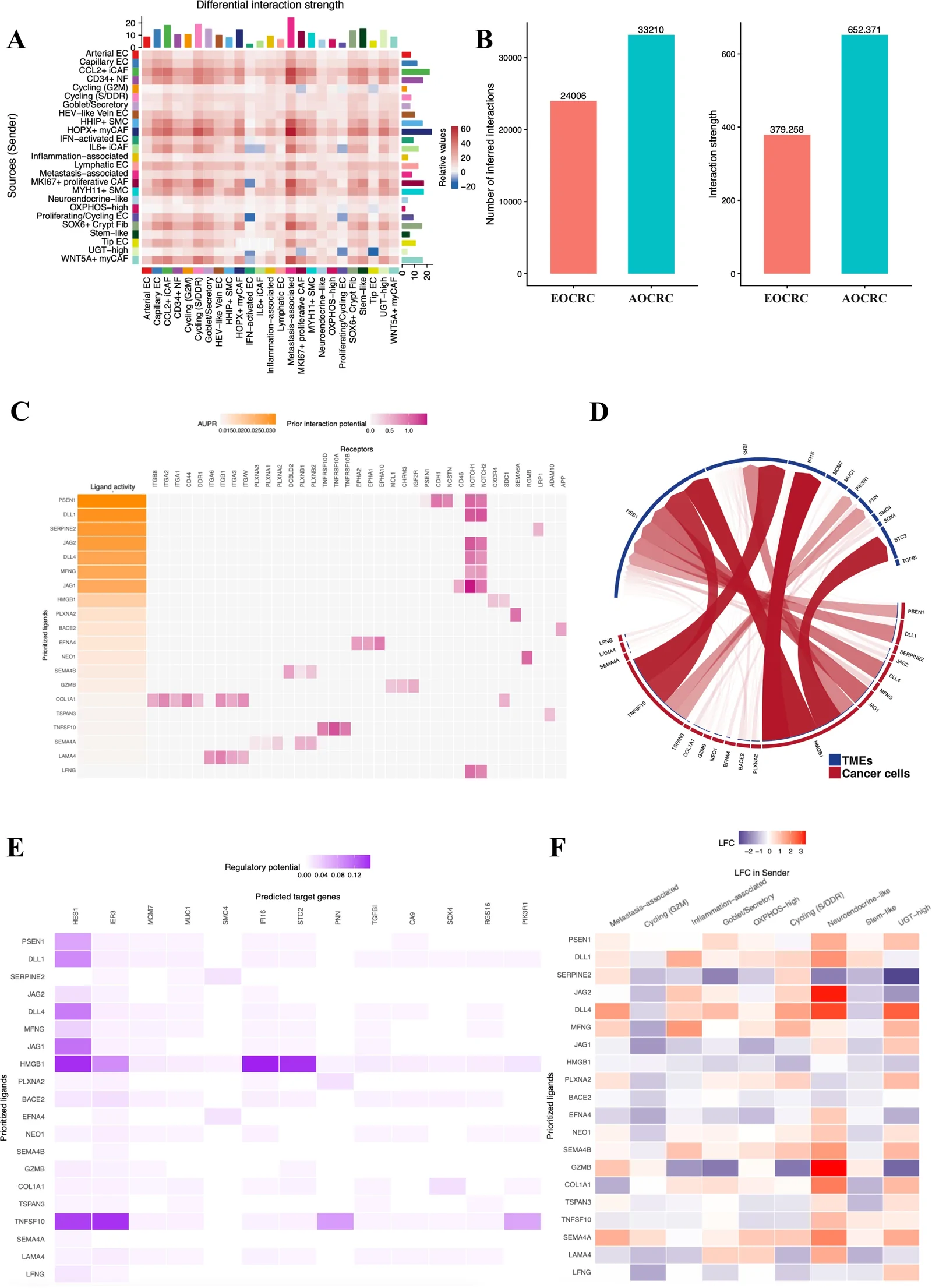
NOTCH-associated intercellular communication network in EOCRC and AOCRC. **(A)** Heatmap illustrating the differential cell-cell interaction strength between cell populations in EOCRC versus AOCRC. **(B)** Bar plots comparing the total number and overall strength of inferred interactions between EOCRC and AOCRC. **(C)** Heatmap displaying EOCRC-upregulated cancer cell-derived ligands prioritized by NicheNet ligand activity (AUPR) and their prior interaction potential with cognate receptors expressed in TME cells. **(D)** Chord diagram illustrating the inferred interactions between EOCRC-upregulated cancer cell-derived prioritized ligands and their predicted downstream target genes in TME cells. **(E)** Heatmap showing the regulatory potential of EOCRC-upregulated cancer cell-derived prioritized ligands on their predicted downstream target genes in TME cells. **(F)** Heatmap depicting the differential expression (log fold change) of prioritized ligands across cancer cell populations in EOCRC versus AOCRC.

To examine signals originating from malignant epithelial cells, genes upregulated in EOCRC cancer cells were evaluated using NicheNet for their potential to regulate transcriptional responses in tumor-microenvironmental recipient cells. This analysis prioritized several candidate signaling molecules and pathway-associated genes, including PSEN1, DLL1, EFNB2, JAG2, DLL4, MFNG, JAG1, HMGB1, PLXNA2, BACE2, NEO1, SEMA4B, GZMB, COL1A1, TSPAN3, TNFSF10, SEMA4A, LAMA4, and LFNG (**Figure 6C**). Their predicted receptor associations indicated that multiple signaling systems, including NOTCH-related, ephrin, semaphorin, extracellular matrix, and inflammatory pathways, may contribute to malignant-cell-to-microenvironment communication.

Ligand-target inference further connected these prioritized signals with candidate transcriptional responses in recipient stromal and endothelial cells (**Figures 6D, E**). Among the predicted targets were genes related to NOTCH signaling, cellular stress responses, extracellular matrix regulation, and proliferation-associated processes. In particular, HES1, a canonical transcriptional readout of NOTCH activity, was identified among the predicted downstream targets. However, these relationships represent computationally inferred regulatory links and do not establish direct ligand-induced activation.

The expression patterns of the prioritized sender-associated genes varied substantially across malignant epithelial states (**Figure 6F**). Several candidates showed increased expression in Cycling (S/DDR), Cycling (G2M), Stem-like, or other malignant epithelial populations in EOCRC relative to AOCRC, whereas others displayed subtype-specific decreases. This heterogeneity suggests that cancer-cell-derived communication in EOCRC is distributed across distinct epithelial states rather than being restricted to a single uniformly activated population. Collectively, these results identify selective remodeling of malignant-cell-to-stromal communication in EOCRC and nominate NOTCH-associated signaling as one component of a broader intercellular network.

### 3.7 Stromal and endothelial signals converge on inflammation-associated malignant epithelial cells

The reciprocal influence of the TME on malignant epithelial cells was subsequently examined by prioritizing ligands expressed by fibroblast and endothelial populations that could regulate inflammation-associated malignant epithelial cells. NicheNet analysis identified DLL1, FGF2, SERPINE2, PSEN1, JAG1, SEMA6B, NEO1, COL4A1, HMGB1, MXRA5, GPC3, DUSP18, EFNA4, COL13A1, RGMA, BACE2, TSPAN3, TNFSF10, and EFNA1 among the highest-ranked candidates (**Figure 7A**). These candidates represented several signaling classes, including NOTCH-associated ligands, growth factors, extracellular matrix components, axon-guidance molecules, and stress-related mediators.

**Figure 7:**
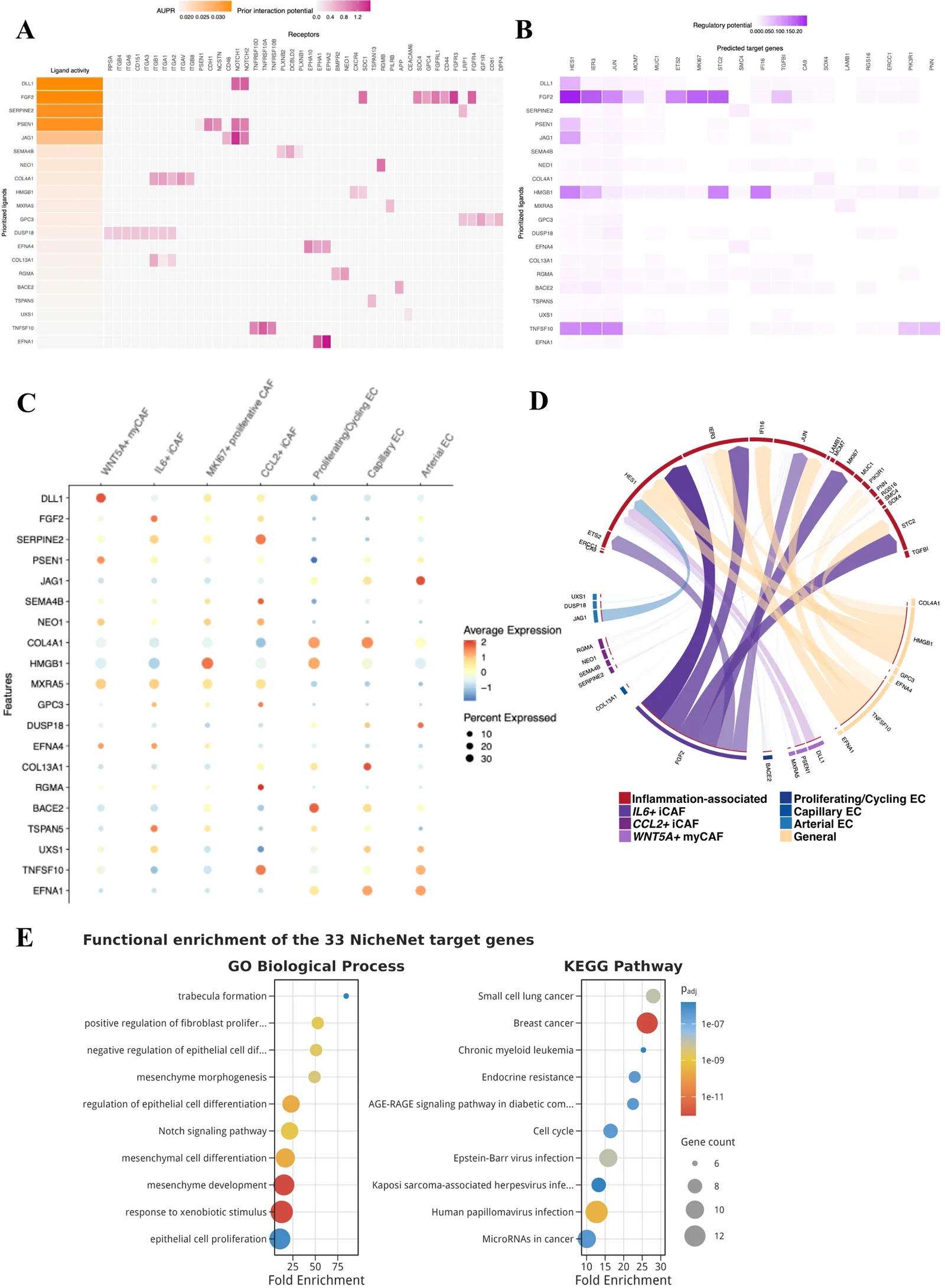
NOTCH-associated intercellular signaling in EOCRC. **(A)** Heatmap showing prioritized TME-derived ligands upregulated in EOCRC relative to AOCRC, ranked by NicheNet ligand activity (AUPR), and their interaction potential with cognate receptors expressed in inflammation-associated tumor cells, with NOTCH-related ligands highlighted. **(B)** Heatmap showing the regulatory potential of prioritized ligands on their predicted downstream target genes. **(C)** Dot plot illustrating the expression patterns of selected prioritized ligands across diverse stromal and endothelial cell populations within the EOCRC versus AOCRC. **(D)** Chord diagram showing inferred ligand–target interactions from TME to inflammation-associated tumor cells. **(E)** Functional enrichment analysis of the 33 NicheNet-prioritized target genes. Gene Ontology (GO) Biological Process enrichment analysis is shown on the left, and Kyoto Encyclopedia of Genes and Genomes (KEGG) pathway enrichment analysis is shown on the right.

Predicted ligand–target analysis indicated that the prioritized microenvironmental signals could converge on transcriptional programs associated with NOTCH signaling, cellular stress, extracellular matrix remodeling, and tumor-cell state regulation (**Figure 7B**). DLL1 and JAG1 were linked to predicted NOTCH-responsive targets, whereas FGF2 showed broad regulatory potential across multiple candidate genes. These computational associations support the presence of a multicellular signaling network but do not by themselves demonstrate direct activation of proliferation, survival, or invasion.

The cellular sources of these candidate signals were heterogeneous across fibroblast and endothelial subsets (**Figure 7C**). WNT5A+ myCAFs, IL6+ iCAFs, MKI67+ proliferative CAFs, CCL2+ iCAFs, proliferating/cycling ECs, capillary ECs, and arterial ECs each contributed distinct combinations of prioritized genes. For example, DLL1 and FGF2 were detectable in selected CAF populations, whereas COL4A1, HMGB1, BACE2, and other candidates showed broader or endothelial-enriched expression patterns. These findings suggest that no single stromal subset exclusively accounts for the inferred signaling network; rather, multiple CAF and endothelial populations may jointly shape the transcriptional state of inflammation-associated malignant epithelial cells. The chord diagram further summarized predicted connections between microenvironment-derived signals and downstream genes in malignant epithelial recipient cells (**Figure 7D**). Overall, the analysis supports a bidirectional communication model in which malignant epithelial cells influence the TME while CAF and endothelial populations provide reciprocal signals to malignant cells.

To substantiate the mechanistic role of these prioritized signals, we performed functional enrichment of the 33 NicheNet target genes, which revealed significant over-representation of the NOTCH signaling pathway (*P* = 9 × 10^-6^), epithelial and fibroblast proliferation, epithelial-to-mesenchymal transition, and cell-cycle regulation (**Figure 7E**). To assess whether these NOTCH-associated relationships are robust to donor composition, we correlated patient-level stromal ligand expression with the malignant 33-gene target score across the full cohort and each leave-one-cohort-out fold (**Supplementary Figure S4B**). DLL4 (r = 0.29–0.40) and JAG1 (r = 0.01–0.32) remained positively and significantly correlated with the target program in all folds, whereas DLL1 and FGF2 were not robust, identifying DLL4 and JAG1 as the most donor-consistent NOTCH-associated ligands.

### 3.8 The prognostic association of NOTCH pathway activity differs by age group

The 33 NicheNet-prioritized target genes were ranked according to the strength and consistency of their age-dependent prognostic interactions in Cox regression models across both the TCGA-COADREAD and GSE39582 cohorts to derive a more robust and biologically interpretable prognostic signature. The five genes showing the strongest concordant gene–age interactions were retained, yielding a five-gene NOTCH–epithelial–mesenchymal transition (EMT)-associated panel comprising HEY1, ZEB1, TWIST1, VIM, and FN1 (**Figure 8A**). Higher panel expression, modeled as a continuous variable per standard-deviation increase, was significantly associated with poorer OS in patients with EOCRC in both cohorts (TCGA-COADREAD: hazard ratio (HR) = 3.43, 95% CI = 1.44–8.14; GSE39582: *HR* = 2.09, 95% CI = 1.31–3.34, *P <0.01*), whereas no significant association was observed in AOCRC (*HR* = 1.09, *P >*0.05; **Figure 8C**). Consistently, the interaction between panel expression and age group was significant in both datasets (*P <0.01*; **Figure 8B**). Collectively, these findings identify a NOTCH–EMT-associated transcriptional program whose prognostic association appears preferentially enriched in EOCRC. Given that the gene panel was derived and evaluated within the same two cohorts, its prognostic utility requires validation in independent prospective datasets. Consistent with a survival-derived rather than expression-derived signal, patient-level expression of the five panel genes did not differ between EOCRC and AOCRC (*P* = 0.835), and the patient-level correlation between the stromal NOTCH-ligand score and panel expression was weak and non-significant (**Supplementary Figure S4C**), indicating that the panel’s prognostic association arises from the gene × age-group interaction rather than from age-enriched NOTCH–EMT expression.

**Figure 8:**
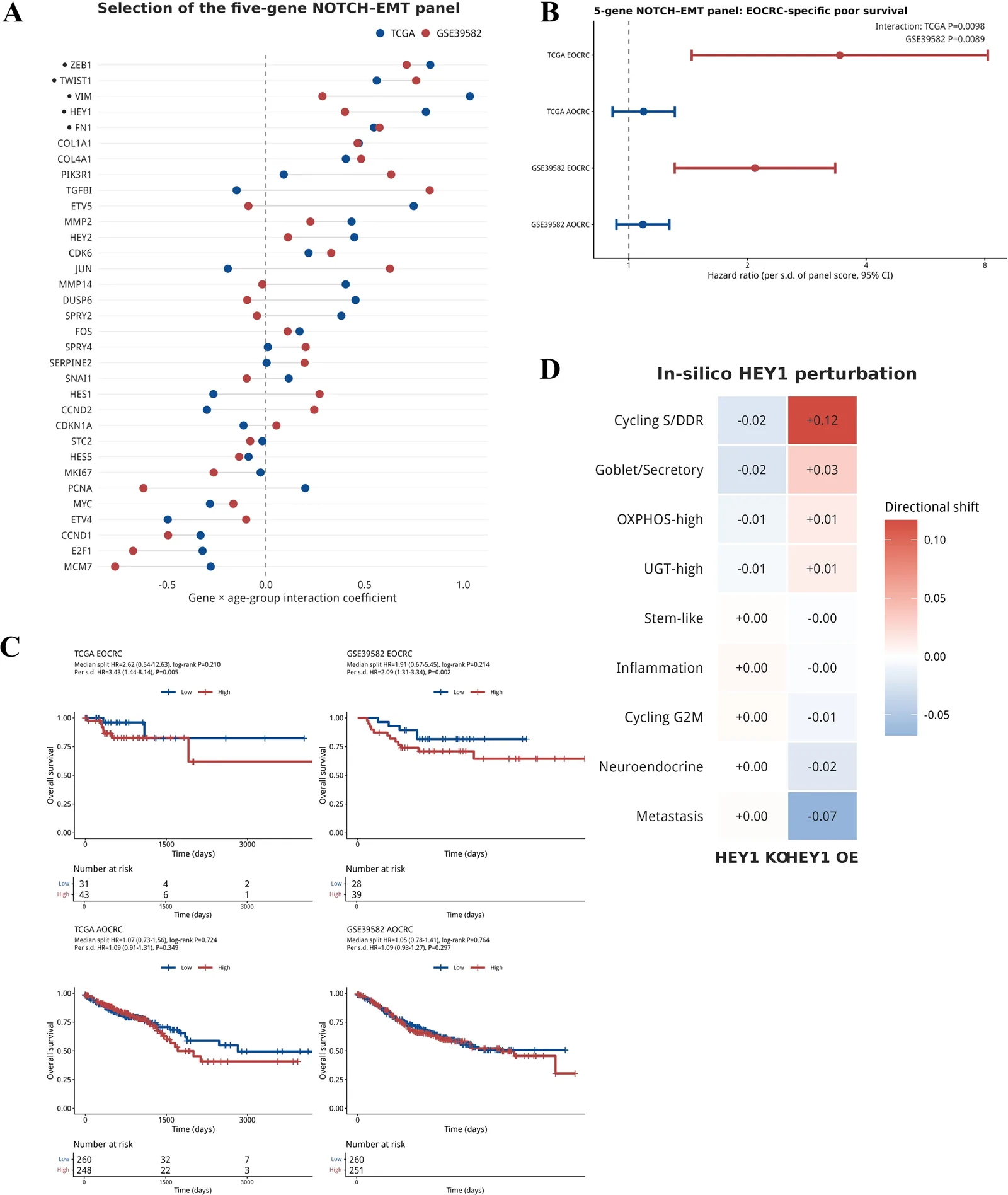
Development and prognostic evaluation of a five-gene NOTCH–EMT-associated panel and *in silico* perturbation of HEY1. **(A)** Ranking of the 33 NicheNet-prioritized target genes according to the strength of their gene × age-group interaction coefficients in Cox regression models across the TCGA-COADREAD and GSE39582 cohorts. **(B)** Forest plot showing the age-stratified prognostic effects of the five-gene NOTCH–EMT-associated panel in the TCGA-COADREAD and GSE39582 cohorts. Hazard ratios and 95% confidence intervals were estimated using Cox proportional hazards models with panel expression modeled as a continuous variable per standard-deviation increase. The panel × age-group interaction was significant in both cohorts. **(C)** Kaplan–Meier survival curves for overall survival according to high versus low five-gene panel expression in EOCRC and AOCRC patients from the TCGA-COADREAD and GSE39582 cohorts. Risk tables are shown below each plot. **(D)** CellOracle-based in silico perturbation analysis of HEY1 across malignant epithelial states. Heatmap values indicate the directional shift along the predefined proliferative–differentiated state axis following simulated HEY1 knockout or overexpression.

To further examine the regulatory contribution of the NOTCH effector HEY1, the only canonical NOTCH-responsive transcriptional regulator retained in the five-gene panel, *in silico* perturbation analysis was performed in malignant epithelial cells using CellOracle. Simulated HEY1 overexpression preferentially shifted the Cycling (S/DDR) population toward the proliferative end of the predefined state axis (directional shift = +0.12), whereas HEY1 knockout induced comparatively modest changes (**Figure 8D**). These results provide computational evidence that increased HEY1 activity may reinforce the proliferative transcriptional state enriched in EOCRC, supporting a potential role for downstream NOTCH signaling in maintaining this malignant cell-state program.

## 4 Discussion

EOCRC has emerged as a major clinical challenge, yet the biological basis for its distinct and often more aggressive behavior remains insufficiently understood. In the clinic, EOCRC is more frequently associated with distal or rectal location, advanced stage at presentation, and adverse histological features (2, 30). However, whether these clinical differences reflect unique biological mechanisms or simply represent an earlier manifestation of conventional CRC remains incompletely understood.

By integrating large-scale single-cell transcriptomic datasets from 174 patients, the present study provides a cellular framework demonstrating that EOCRC is characterized by coordinated remodeling across malignant epithelial, endothelial, and fibroblast compartments. At the malignant level, EOCRC was characterized by an increased representation of inflammation-associated and cycling epithelial states, accompanied by the coordinated expansion of proliferative populations across endothelial and fibroblast compartments. These findings suggest that the biological differences associated with early-onset disease are not confined to tumor-intrinsic epithelial alterations but are distributed across multiple interacting cellular lineages. This observation is consistent with the emerging view that clinically relevant tumor heterogeneity is organized through multicellular states and niches rather than individual malignant populations alone (10, 31).

The enrichment of Inflammation-associated and Cycling (S/DDR) malignant epithelial states in EOCRC further indicates that age-associated differences are reflected in the relative occupancy of distinct epithelial transcriptional programs. Previous single-cell studies have demonstrated extensive epithelial-state heterogeneity in CRC, including stem-like, proliferative, inflammatory, metabolic, and differentiated programs (20, 32). In particular, Joanito et al. showed that intrinsic epithelial states represent a major dimension of CRC heterogeneity that is partly independent of MSI status and stromal context. The present findings extend this framework by suggesting that the distribution of these malignant states may also vary according to age at disease onset.

A notable finding of this study is that the distinct epithelial phenotypes observed in EOCRC were not accompanied by substantial differences in chromosomal instability after accounting for MSI status. Although previous studies have suggested age-associated differences in genomic alterations, our comprehensive CNV analysis using multiple burden metrics demonstrated that MSI status, rather than age itself, was the predominant determinant of CNV variation. Notably, this result differs from a recent integrated single-cell analysis reporting increased CNV burden in younger patients with CRC (33). Several factors may contribute to this discrepancy, including differences in cohort composition, age-group definition, MSI distribution, CNV-inference strategy, and covariate adjustment. In the present analysis, the apparent age-associated differences were markedly attenuated after adjustment for MSI status and were not robust in stratified sensitivity analyses. These observations suggest that increased chromosomal instability should not be considered a universal feature of EOCRC and highlight the importance of accounting for MSI composition when comparing genomic instability across age groups. This observation provides an important mechanistic implication. If broad chromosomal alterations are insufficient to explain the divergent transcriptional states between EOCRC and AOCRC, then additional regulatory layers—including epigenetic regulation, cellular plasticity, and microenvironmental signaling—are likely to play dominant roles in shaping EOCRC biology. Increasing evidence indicates that tumor cell states can undergo substantial phenotypic transitions without major changes in genomic architecture, driven instead by transcriptional programs and extracellular cues (34, 35). Therefore, the absence of a robust age-associated CNV signature redirects attention toward transcriptional plasticity and microenvironmental regulation as potential contributors to EOCRC-associated phenotypes.

Beyond malignant epithelial cells, our analysis revealed extensive remodeling of endothelial and fibroblast compartments in EOCRC. Specifically, EOCRC displayed increased proportions of proliferating/cycling ECs and MKI67+ proliferative CAFs, together with altered iCAF states. These findings suggest that EOCRC is associated with a TME enriched for cellular programs involved in proliferation, inflammatory regulation, and tissue remodeling. CAF heterogeneity has increasingly been recognized as a major determinant of CRC progression. Different CAF subsets can regulate tumor growth, immune exclusion, extracellular matrix organization, and therapeutic response through distinct signaling programs (12, 13). Similarly, EC states have emerged as active regulators of tumor progression rather than passive structural components. Tumor-associated endothelial populations can modulate angiogenesis, immune recruitment, and metastatic potential through context-dependent transcriptional programs. Our identification of proliferative CAF and endothelial states in EOCRC therefore supports a model in which younger-onset tumors develop within a uniquely activated microenvironment. Importantly, these findings suggest that EOCRC aggressiveness may arise from coordinated multicellular amplification rather than from malignant epithelial alterations alone.

Through CellChat and NicheNet analyses, we identified selective remodeling of epithelial–stromal communication networks in EOCRC. Although AOCRC exhibited greater overall numbers and strength of inferred interactions, EOCRC showed distinct enrichment of specific communication pathways involving malignant epithelial cells, CAFs, and endothelial populations. Among these pathways, NOTCH-associated signaling emerged as a recurrent candidate regulator. NOTCH signaling is a highly conserved pathway involved in cell fate determination, stemness maintenance, proliferation, and microenvironmental communication. In CRC, NOTCH activation has been implicated in maintaining cancer stem-like populations and regulating tumor differentiation states (36). Our data extend these observations by suggesting that NOTCH-associated signaling may contribute to age-dependent differences in CRC biology through coordinated interactions between malignant cells and stromal populations. Importantly, our results do not indicate that NOTCH activation alone defines EOCRC. Instead, the data support a model in which NOTCH-associated ligands, including DLL1, DLL4, JAG1, and JAG2, participate within a broader signaling network involving growth factors, extracellular matrix regulators, and inflammatory mediators. This interpretation is supported by recent experimental evidence showing that endothelial-derived JAG1 can activate NOTCH signaling in CRC cells and promote stemness and tumor progression, providing biological support for endothelial-to-epithelial NOTCH communication in CRC (37). Nevertheless, the CellChat and NicheNet associations identified here remain computational predictions and do not establish direct ligand–receptor engagement. Spatial colocalization, protein-level validation, and perturbation of specific DLL/JAG–NOTCH interactions will therefore be required to establish functional causality. Donor- and cohort-level sensitivity analyses (leave-one-cohort-out and donor-balanced resampling; **Supplementary Figure S4**) further indicated that the most robust NOTCH-associated signal is the NicheNet-derived DLL4/JAG1-target relationship, whereas NOTCH information flow and ligand/receptor expression were not themselves age-dependent, reinforcing the interpretation that NOTCH signaling is one candidate component of a broader remodeled network rather than a dominant EOCRC-specific driver.

The prognostic analyses further connected the NOTCH-associated communication network with a downstream mesenchymal transcriptional program. A five-gene panel comprising HEY1, ZEB1, TWIST1, VIM, and FN1 showed a stronger association with poor OS in EOCRC than in AOCRC across both the TCGA-COADREAD and GSE39582 cohorts. The biological composition of this panel is consistent with a NOTCH–EMT regulatory axis: HEY1 is a canonical downstream effector of NOTCH signaling, whereas ZEB1 and TWIST1 are major EMT-associated transcription factors and VIM and FN1 are established components of mesenchymal cell states (38, 39). Together with the enrichment of proliferation- and EMT-related processes among the NicheNet target genes, these findings suggest that NOTCH-associated intercellular signaling may converge on proliferative and mesenchymal malignant programs in EOCRC. However, because the five genes were selected according to age-dependent prognostic interactions observed in the same two cohorts used for subsequent evaluation, the resulting panel should be regarded as a biologically informed candidate signature rather than a validated prognostic biomarker. Independent prospective validation will be required to establish its reproducibility and clinical utility.

The CellOracle analysis provided an additional computational link between NOTCH signaling and malignant epithelial state regulation. Simulated HEY1 overexpression preferentially shifted the Cycling (S/DDR) population toward the proliferative pole, whereas HEY1 knockout produced comparatively modest changes. Given that CellOracle infers state responses from gene-regulatory networks rather than experimentally manipulating gene activity (29), these findings should not be interpreted as evidence that HEY1 is required for maintenance of the EOCRC phenotype. Instead, they support the hypothesis that increased HEY1 activity may reinforce the proliferative transcriptional state enriched in EOCRC. Genetic or pharmacological perturbation of HEY1 and upstream NOTCH components in patient-derived organoid or co-culture models will be necessary to establish causality.

The rapid birth-cohort-associated increase in EOCRC incidence has prompted growing interest in environmental, metabolic, dietary, and microbiome-related exposures that cannot be explained by inherited susceptibility alone (40–42). Recent methodological advances, including the prospective cohort incident-tumor biobank method (PCIBM), provide a powerful framework for studying cancer etiology by capturing molecular alterations before and at tumor initiation (43, 44). The PCIBM strategy has been proposed as an important future direction for identifying early biological changes associated with cancer development and overcoming limitations of retrospective tumor-based studies (e.g., Unique role of the prospective cohort incident-tumor biobank method in etiological research). Such approaches may be particularly valuable for EOCRC, where changing environmental exposures, lifestyle transitions, microbiome alterations, and immune–metabolic factors may interact with host susceptibility over time. Future studies integrating prospective cohorts with spatial transcriptomics, proteomics, microbiome profiling, and functional perturbation models will be necessary to determine whether the NOTCH-associated communication networks identified here represent therapeutic vulnerabilities. Moreover, experimental validation using organoid-CAF-endothelial co-culture systems and *in vivo* models will be critical to establish whether disrupting these multicellular interactions can alter EOCRC progression.

Several limitations should be considered. First, although integration of multiple public scRNA-seq datasets increases statistical power, differences in sequencing platforms, patient selection, and clinical annotation may introduce residual confounding despite batch correction and covariate adjustment. Second, the number of EOCRC samples remains limited, reflecting the relatively lower incidence of this disease subtype. Larger independent cohorts will be required to validate the identified cellular states and signaling programs. Third, ligand–receptor inference methods such as CellChat and NicheNet rely on transcriptomic measurements and prior biological knowledge; therefore, predicted interactions require validation at the protein level and through functional experiments. Finally, although the association between NOTCH activity and EOCRC outcome suggests clinical relevance, prospective validation and therapeutic studies are needed before translation into clinical applications.

In summary, this study establishes a single-cell framework explaining the biological distinctiveness of EOCRC. Rather than being defined by increased chromosomal instability, EOCRC appears to arise from coordinated remodeling of malignant epithelial states, vascular niches, and fibroblast populations. The identification of age-associated epithelial–stromal communication networks, particularly NOTCH-associated signaling interactions, provides a mechanistic basis for understanding EOCRC heterogeneity and highlights potential prognostic associations warranting functional validation in independent cohorts.

## Data Availability

The original contributions presented in the study are included in the article/**Supplementary Material**. Further inquiries can be directed to the corresponding authors.

## Supplementary material

Supplementary Figure 1 Batch correction validation and quality control. **(A)** PCA of the merged dataset before Harmony integration, colored by original dataset, showing strong dataset-specific clustering prior to batch correction. **(B)** UMAP of the integrated dataset after Harmony batch correction, colored by original dataset, demonstrating effective mixing of all seven cohorts. **(C)** UMAP split by age group (EOCRC, red; AOCRC, blue), showing balanced spatial distribution after integration. **(D)** Cell type composition bar plot by dataset, confirming consistent representation of all nine major cell lineages across the seven integrated cohorts. **(E)** Batch mixing entropy per cluster, quantifying the uniformity of dataset representation within each cell type. **(F–H)** Quality control metrics (number of detected genes, total UMI counts, and percentage of mitochondrial reads) across datasets before and after filtering. **(I)** Number of high-quality cells retained per patient across the seven cohorts after quality control filtering.

Supplementary Figure 2 CNV sensitivity analysis. **(A)** Per-arm CNV differences stratified by MSI status (MSI-H, MSS). Each bar represents the EOCRC − AOCRC mean CNV difference for one chromosome arm within each MSI stratum. Black bars, EOCRC-biased; gray bars, AOCRC-biased. **(B)** Per-arm CNV differences stratified by clinical stage (T1, T2, T3, T4). **(C)** Per-arm CNV differences stratified by tumour sidedness (left, right). **(D–F)** Scatter plots comparing overall ΔCNV (x-axis) versus stratum-specific ΔCNV (y-axis) for MSI status **(D)**, clinical stage **(E)**, and tumour sidedness **(F)**. Each point represents one chromosome arm. Pearson correlation coefficients and linear regression fits are annotated per stratum. Dashed lines indicate y = 0 and x = 0.

Supplementary Figure 3 K-means cluster-count selection and robustness of malignant-cell assignment. **(A)** Within-cluster sum of squares (WSS, blue) and mean silhouette (red) for k = 3–10, with k = 7 marked by a dashed line. WSS decreases monotonically without a sharp elbow, and silhouette shows a modest local maximum, supporting k = 7 as a balance between capturing distinct CNV archetypes and over-fragmentation. **(B)** Percentage of malignant epithelial cells across k = 5–9, with the normal-like cluster defined as the one with the lowest mean CNV fraction. The malignant-cell assignment is stable (~53–57%) across k, and the k = 7 assignment (red) is concordant with independent inferCNV calls.

Supplementary Figure 4 Robustness analyses of NOTCH signaling and its association with five-gene panel. **(A)** CellChat-derived NOTCH relative information flow in AOCRC and EOCRC using the full donor-balanced dataset and leave-one-cohort-out (LOCO) analyses. **(B)** LOCO robustness of NicheNet-predicted ligand-target relationships. Relationships consistently supported in all eight analyses are classified as robust (8/8, blue), whereas less consistently supported relationships are classified as weak (gray). **(C)** Patient-level expression and NOTCH-ligand associations of a five-gene panel comprising HEY1, ZEB1, TWIST1, VIM, and FN1. The left panel shows mean expression in AOCRC and EOCRC; no significant overall group difference was observed after multiple-testing adjustment (*P* = 0.835). The right panel shows the median correlation between the NOTCH-ligand score and individual panel genes across LOCO analyses, with horizontal lines indicating the range; none of the correlations was significant across folds (*P*>0.05).
